# A serum-stable RNA aptamer specific for SARS-CoV-2 neutralizes viral entry

**DOI:** 10.1073/pnas.2112942118

**Published:** 2021-12-07

**Authors:** Julián Valero, Laia Civit, Daniel M. Dupont, Denis Selnihhin, Line S. Reinert, Manja Idorn, Brett A. Israels, Aleksandra M. Bednarz, Claus Bus, Benedikt Asbach, David Peterhoff, Finn S. Pedersen, Victoria Birkedal, Ralf Wagner, Søren R. Paludan, Jørgen Kjems

**Affiliations:** ^a^Interdisciplinary Nanoscience Center, Aarhus University DK-8000 Aarhus, Denmark;; ^b^Centre for Cellular Signal Patterns (CellPAT), Aarhus University, Aarhus DK-8000, Denmark;; ^c^Department of Molecular Biology and Genetics, Aarhus University, DK-8000 Aarhus, Denmark;; ^d^Department of Biomedicine, Aarhus University DK-8000 Aarhus, Denmark;; ^e^Department of Chemistry, Aarhus University, DK-8000, Aarhus, Denmark;; ^f^Institute of Medical Microbiology and Hygiene/Molecular Microbiology (Virology), Regensburg University 93053 Regensburg, Germany;; ^g^Institute of Clinical Microbiology and Hygiene, University Hospital Regensburg, Regensburg 93053, Germany

**Keywords:** aptamer selection, SARS-CoV-2 targeting, aptamer multimerization, viral neutralization, spike protein

## Abstract

The severe acute respiratory syndrome coronavirus 2 (SARS-CoV-2) pandemic has created an urgent need for new technologies to treat COVID-19. Here we report a 2′-fluoro protected RNA aptamer that binds with high affinity to the receptor binding domain (RBD) of SARS-CoV-2 spike protein, thereby preventing its interaction with the host receptor ACE2. A trimerized version of the RNA aptamer matching the three RBDs in each spike complex enhances binding affinity down to the low picomolar range. Binding mode and specificity for the aptamer–spike interaction is supported by biolayer interferometry, single-molecule fluorescence microscopy, and flow-induced dispersion analysis in vitro. Cell culture experiments using virus-like particles and live SARS-CoV-2 show that the aptamer and, to a larger extent, the trimeric aptamer can efficiently block viral infection at low concentration. Finally, the aptamer maintains its high binding affinity to spike from other circulating SARS-CoV-2 strains, suggesting that it could find widespread use for the detection and treatment of SARS-CoV-2 and emerging variants.

## Significance

Developing molecules capable of binding to SARS-CoV-2 spike protein and inhibiting viral infection is of utmost importance for the detection and therapy of COVID-19. We have developed and engineered a serum-stable RNA aptamer specific for SARS-CoV-2 spike protein. We further show that scaffolding three aptamers together increases the binding efficiency to the low picomolar range and enables very efficient neutralization of SARS-CoV-2 infection in cells. The aptamer also shows high affinity for spike protein from variants of concern. Due to its small size and chemical stability, our aptamer holds potential as an alternative to antibodies and nanobodies targeting spike protein.

The severe acute respiratory syndrome coronavirus 2 (SARS-CoV-2) pandemic in 2020–2021 has launched a global quest to find new molecular tools for the detection of the virus and treatment of the potential deadly disease it causes, COVID-19. Despite the exceptional efforts worldwide for containment and unprecedented technological progress in vaccine development, the challenge to find an effective cure remains, due to the limited access to SARS-CoV-2 vaccines, particularly in developing countries, and the emergence of new viral strains that can evade immune responses and potentially compromise the efficacy of current vaccines. Therefore, it is of utmost importance to focus efforts on developing affordable and easy-to-produce antiviral molecules against SARS-CoV-2 infection.

Like other coronaviruses, SARS-CoV-2 expresses a surface spike (S) glycoprotein which is composed of two domains (S1 and S2) (1) and forms a trimeric structure capable of interacting with human cells (2, 3). In particular, the receptor binding domain (RBD) located on the S1 subunit of the spike protein binds with high affinity to human angiotensin-converting enzyme 2 (ACE2) (4, 5), which, in conjunction with the associated transmembrane protease, serine 2 (TMPRSS2), facilitates viral uptake. Efforts to neutralize viral infection have therefore mainly focused on inhibiting the spike–ACE2 interaction. Antibodies (Abs) have been developed and are currently used for SARS-CoV-2 detection, and some, primarily those targeting RBD, show therapeutic potential due to their potent neutralizing effect (6–13). However, the high costs of Ab production, the use of animals to generate them, and their poor stability at ambient temperatures remain a disadvantage. Moreover, Ab immunogenicity and the risk of Ab-dependent enhancement of infection associated with Fc-containing Abs put their therapeutic potential at risk (14).

V_H_H Abs or nanobodies raised to the spike protein may overcome some of these drawbacks (15–20) but are more prone to immunological response (21, 22). Interesting alternatives such as de novo proteins based on the host ACE2 receptor (23) and other synthetic molecules (24) have been investigated and may, if potential immunogenicity and stability problems are solved, help develop efficient detection methods and drugs.

Nucleic acid-based aptamers have gained increased attention as alternatives to Abs due to their ease of production, low immunogenicity, high thermal and chemical stability, and smaller size, while they still retain comparable target binding and specificity. Aptamers are short single-stranded oligonucleotides, developed through an in vitro selection process termed SELEX (systematic evolution of ligands by exponential enrichment), that bind with high affinity and selectivity to cognate targets (25–27). During the last few decades, a wide variety of aptamers binding to diverse biologically relevant targets (28), including viruses (29, 30), have been identified. However, selection of aptamers targeting spike protein has proven difficult. An explanation for this may be that highly glycosylated proteins such as SARS-CoV-2 spike are challenging to target with nucleic acid-based binders. Indeed, to date, there are only a few reports on DNA aptamers targeting SARS-CoV-2 spike where the authors report leading aptamers with affinities in the nanomolar range (31–35).

Here we report the selection and characterization of a serum-stable RNA aptamer, RBD-PB6, that binds with nanomolar affinity to the RBD of SARS-CoV-2 spike protein and neutralizes viral infectivity. The aptamer contains 2′-fluoro pyrimidine modifications to increase its chemical stability and resistance to nucleases (36, 37), and it shows high selectivity to SARS-CoV-2 and related strains, including alpha and beta. Aptamer multimerization strongly enhances its affinity to the picomolar range as well as its SARS-CoV-2 neutralizing potency. These unique features open avenues for developing inexpensive, fast, and reliable detection platforms for SARS-CoV-2 and therapeutic application for COVID-19.

## Results

### Selection, Sequencing, and Truncation Studies of RBD-PB6.

**Fig. 1.**
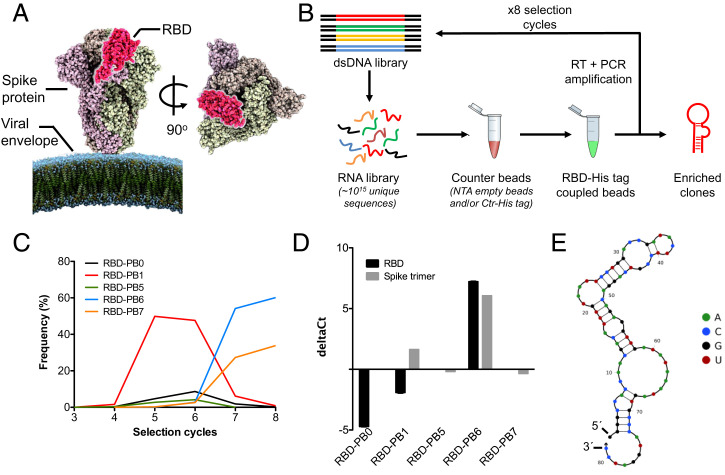
Selection of an RNA aptamer binding to RBD. (*A*) Side and top view of spike protein trimer (Protein Data Bank ID code 6VXX) with one of the three RBDs highlighted in magenta. (*B*) SELEX scheme for the selection of aptamers against RBD. (*C*) NGS data showing the abundance (frequency %) of the most representative RNA clones during selection. (*D*) The qPCR binding assay of five selected clones to RBD and spike protein immobilized on magnetic beads. DeltaCt values are calculated by subtracting the cycle threshold (Ct) obtained from pull-down assays using beads functionalized with either RBD or the full spike protein to control protein [Ct(target) − Ct(control)]. (*E*) Secondary structure of RBD-PB6 predicted by NUPACK.

For the aptamer selection, we targeted the RBD of SARS-CoV-2 spike protein that recognizes and binds the ACE2 receptor (Fig. 1*A*, highlighted in magenta). We performed eight cycles of SELEX against recombinant RBD (amino acids 319 to 532) derived from the wild-type (WT) SARS-CoV-2 spike protein using a 2′-fluoro pyrimidine–modified RNA library (38, 39) encompassing a 36-nt randomized region. For the initial selection cycle, we used 1.7 nmol of the RNA library, which provided a wide sequence diversity with ∼10^15^ unique RNA molecules. His-tagged RBD protein expressed in HEK293 cells was immobilized on Ni nitrilotriacetic acid (Ni-NTA) magnetic beads and incubated with the RNA library in each selection cycle. The RNA bound to the beads was subjected to reverse transcription and PCR amplification. A counterselection step was introduced in all cycles to avoid unspecific binders, using a control His-tagged protein (Ctr-His), and an additional counterselection step with empty Ni-NTA beads was included from the sixth selection cycle (Fig. 1*B*). Iterative cycles of selection while reducing the amount of protein and RNA, as well as the incubation time, yielded enrichment of different clones in the library. Sequencing data show that several clones (RBD-PB0, RBD-PB1, and RBD-PB5) dominated the library pool in early selection cycles, representing up to 50 to 60% of the total amount of sequences. In selection cycle 6, the frequency of earlier enriched sequences drastically decreased, leading to the enrichment of other predominant clones. In particular, RBD-PB6 and RBD-PB7 displayed a strong enrichment in the last selection cycles, representing more than 90% of the library in cycle 8 (Fig. 1*C*).

Enrichment through the different selection cycles was monitored by qPCR and next-generation sequencing (NGS) analysis. Criteria for clone selection was based on frequency and rate of amplification. The relative binding of the selected candidates was assessed by qPCR of RNA recovered in a spike protein pull-down assay. We synthesized individual aptamer clones and incubated them with either RBD-functionalized or full-length trimeric spike-functionalized beads. As a negative control, we used beads functionalized with an unrelated His-tagged control protein (Ctr-His). Despite the initial enrichment of RBD-PB1 and RBD-PB5 and later RBD-PB7 seen during the selection, only the most predominant sequence after the eighth selection cycle, RBD-PB6, showed robust binding to both RBD and trimer spike protein by qPCR (Fig. 1*D*). Fig. 1*E* illustrates the secondary structure of RBD-PB6 predicted by NUPACK.

### RBD-PB6 Binds with High Affinity to SARS-CoV-2 Spike Protein and Blocks Its Interaction with ACE2.

**Fig. 2.**
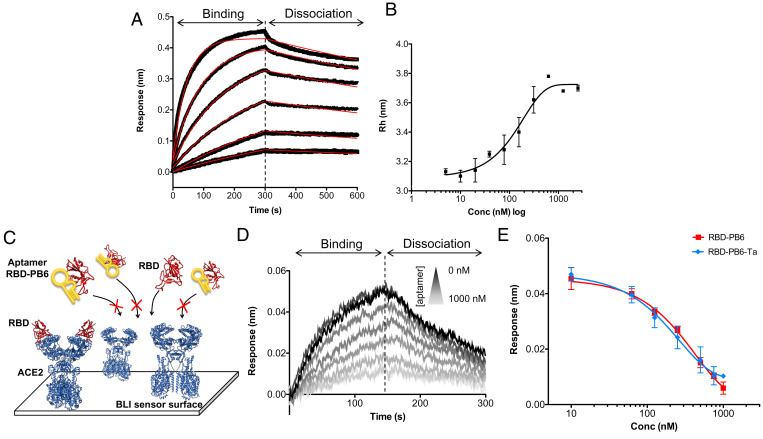
RBD-PB6 binds to RBD and inhibits its interaction with ACE2. (*A*) BLI measuring the binding of RBD-PB6 (in solution) to RBD (immobilized). RBD-PB6 is diluted in 1/2 dilution steps starting from 500 nM (red lines indicate fitting model). (*B*) FIDA experiments showing the increase in hydrodynamic radius in solution upon binding of the RBD to the fluorescently labeled RBD-PB6 (black line indicates best fit to a 1:1 binding model). (*C*) BLI competition assay with ACE2 immobilized on the sensor surface and binding inhibition to RBD at different aptamer concentrations. (*D*) Characteristic wavelength shifts recorded in the competition assays. ACE2 is immobilized on the sensor surface. Then, the sensor is dipped in a solution of RBD preincubated with increasing concentrations of RBD-PB6 (binding phase), and subsequently dipped in washing buffer (dissociation phase). (*E*) Fitted data show the signal decay at increasing concentrations of aptamer used to determine IC_50_ values.

The binding of RBD-PB6 to different SARS-CoV-2 spike constructs was assayed by biolayer interferometry (BLI) and flow-induced dispersion analysis (FIDA) (Fig. 2). To determine the binding affinity of RBD-PB6 to spike protein by BLI, we immobilized His-tagged RBD, spike-S1, full-length spike, or trimeric spike proteins on Ni-NTA–functionalized sensors and tested the binding of RBD-PB6 at increasing concentrations. The BLI measurements showed that RBD-PB6 binds with high affinity (K_D_ ≈ 18 nM) to the RBD alone (Fig. 2*A*) and also interacts with spike-S1 and monomeric and trimeric spike in a stabilized form (*SI Appendix*, Fig. S1 *A* and *B*). These data show that RBD-PB6 interacts with a surface-exposed region of the RBD in the context of the whole trimeric spike protein. Moreover, control experiments with non-His-tagged spike protein or an unrelated His-tagged protein showed that the RBD-PB6 interaction is specific and independent of the protein purification tag used during aptamer selection. For comparison, we performed qualitative binding BLI assays with the lead aptamer (CoV2-RBD-1C) first reported by Song et al. (31) to bind RBD. In our hands, the CoV2-RBD-1C aptamer did not bind specifically to spike protein, and its binding to RBD-related proteins depends on the His-tag. In fact, the previously reported CoV2-RBD-1C aptamer shows significant unspecific binding to our His-tagged control protein, suggesting that the His-tag is the main target (*SI Appendix*, Fig. S1 *C* and *D*). This impedes its application for SARS-CoV-2 intervention or detection.

To test whether our RBD-PB6 aptamer binds spike protein in solution, we performed titration binding experiments by FIDA, which allowed us to monitor changes in hydrodynamic radii of the aptamer–protein complexes at different concentrations and under native binding conditions. The FIDA measurement is based on the accurate quantification of the change in the apparent size (diffusivity) of a labeled molecule (the indicator) binding to a target molecule (the analyte) (40, 41). The apparent indicator and complex sizes are assessed using Taylor dispersion analysis in a capillary under hydrodynamic flow. The change in diffusivity upon complex formation can be used to measure the binding affinity between the analyte and the indicator (*SI Appendix*, Fig. S2). We labeled the aptamer (indicator) with Alexa Fluor 488 fluorophore and incubated it with increasing concentrations of RBD protein (analyte). We found that RBD-PB6 binds to RBD with an estimated K_D_ value of 129 nM, which is about 10 times higher than the BLI measurement (Fig. 2*B*); however, such differences in the K_D_ value between solution- and surface-binding assays are commonly seen in other studies (42). Based on the FIDA data, we can estimate the hydrodynamic radii (Rh) of RBD protein alone to be 2.8 nm, aptamer alone to be ∼3.1 nm, and the resulting complex to be ∼3.8 nm, suggesting an extended area of interaction in the complex, where the aptamer establishes multiple contacts.

To determine whether the binding of RBD-PB6 to spike protein can block the interaction with host ACE2 and potentially prevent viral entry, we performed competition assays using BLI (Fig. 2*C*). To that end, Fc-tagged ACE2 receptor was immobilized on a protein G sensor and incubated with 50 nM RBD protein, preincubated with increasing concentrations of RBD-PB6 aptamer. We found that RBD-PB6 inhibited binding of RBD to ACE2 in a concentration-dependent manner with a concentration that inhibits the response by 50% (IC_50_) of ∼250 nM (Fig. 2 *D* and *E*). This indicates that the RBD-PB6 binding site on RBD overlaps with the binding site for ACE2 and thus holds potential to neutralize SARS-CoV-2 infection.

Based on the predicted secondary structure of RBD-PB6 (Fig. 1*E*), we evaluated a range of truncated versions of the aptamer to increase the atom economy of the binder and to determine the motifs involved in RBD binding (*SI Appendix*, Fig. S3). The truncated aptamers were assayed in a BLI competition experiment with ACE2 (*SI Appendix*, Fig. S4). The full-length 80-nt RBD-PB6 is predicted to form an elongated stem loop which could be shortened to 53 nt (RBD-PB6-Ta) without a loss in binding affinity (IC_50_ value of 200 nM; Fig. 2*E*). Further truncation of the stem loop (RBD-PB6-Tb and RBD-PB6-Tc), shortening of the terminal hairpin loop (RBD-PB6-Td), or base pair covariation in the central part of the stem (RBD-PB6-Tinv) led to complete loss in binding affinity, suggesting that these aptamer motifs/regions are essential for the recognition of SARS-CoV-2 RBD.

### RBD-PB6 Neutralizes SARS-CoV-2 Viral Entry.

**Fig. 3.**
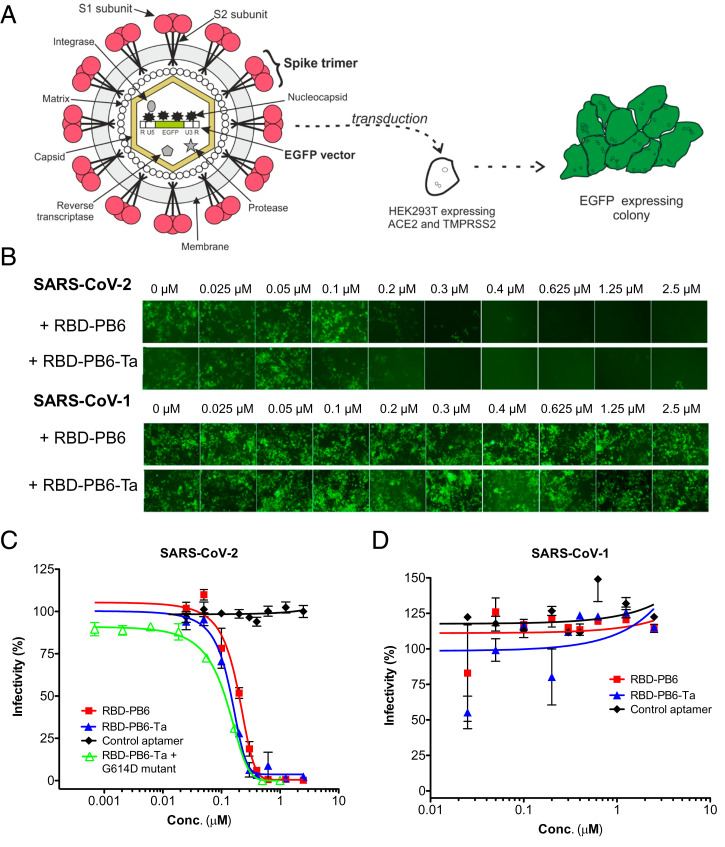
VLP neutralization experiments with SARS-CoV-1 and SARS-CoV-2. (*A*) Schematic representation of a neutralization assay using spike-expressing VLPs. (*B*) Fluorescence microscopy images of HEK293T cells transduced with SARS-CoV-1 or SARS-CoV-2 VLPs carrying an EGFP reporter in the presence of increasing concentrations of aptamer. The size of each image is 704 µm × 528 µm. (*C* and *D*) Flow cytometry analysis of the fluorescence signal for (*C*) SARS-CoV-2 and (*D*) SARS-CoV-1 transduction.

Since RBD-PB6 can block the interaction between recombinant RBD and ACE2, we next performed in vitro neutralization assays using virus-like particles (VLPs). The VLPs are based on inactivated HIV pseudotyped with SARS-CoV-2 spike protein and containing an EGFP vector as a fluorescent reporter (Fig. 3*A*). The target cells (HEK293T cells transfected with plasmids expressing human ACE2 and TMPRSS2) were incubated for 36 h to 48 h with SARS-CoV-2 VLPs and varying amounts of RBD-PB6, RBD-PB6-Ta, and a control aptamer. Subsequently, cells were washed and cultured for 3 d. Fluorescence microscopy shows that increasing concentrations of the aptamer considerably reduce the EGFP signal, indicating that RBD-PB6 efficiently blocks viral uptake (Fig. 3*B*). The fluorescent signal was quantified by flow cytometry, which yielded IC_50_ values of 200 and 140 nM for RBD-PB6 and RBD-PB6-Ta, respectively (Fig. 3*C*), in good correspondence with the BLI in vitro inhibition values. Furthermore, RBD-PB6-Ta neutralized VLPs expressing the D614G spike variant, associated with increased viral infectivity (43), slightly more efficiently than the WT strain, with an IC_50_ value of 110 nM (Fig. 3*C*, green curve).

To investigate the broader specificity of RBD-PB6, we repeated the assay using VLPs pseudotyped with either Middle East respiratory syndrome (MERS) or SARS-CoV-1 spike proteins. No neutralization was observed at any of the concentrations tested against these VLPs (Fig. 3*D* and *SI Appendix*, Fig. S5 for MERS). Thus, the aptamer shows high specificity toward SARS-CoV-2 with no apparent cross-reactivity for MERS and SARS-CoV-1 despite the 75% sequence similarity between RBDs from SARS-CoV-2 and SARS-CoV-1.

### Aptamer Multimerization Improves Binding to Spike and Neutralization Efficacy.

**Fig. 4.**
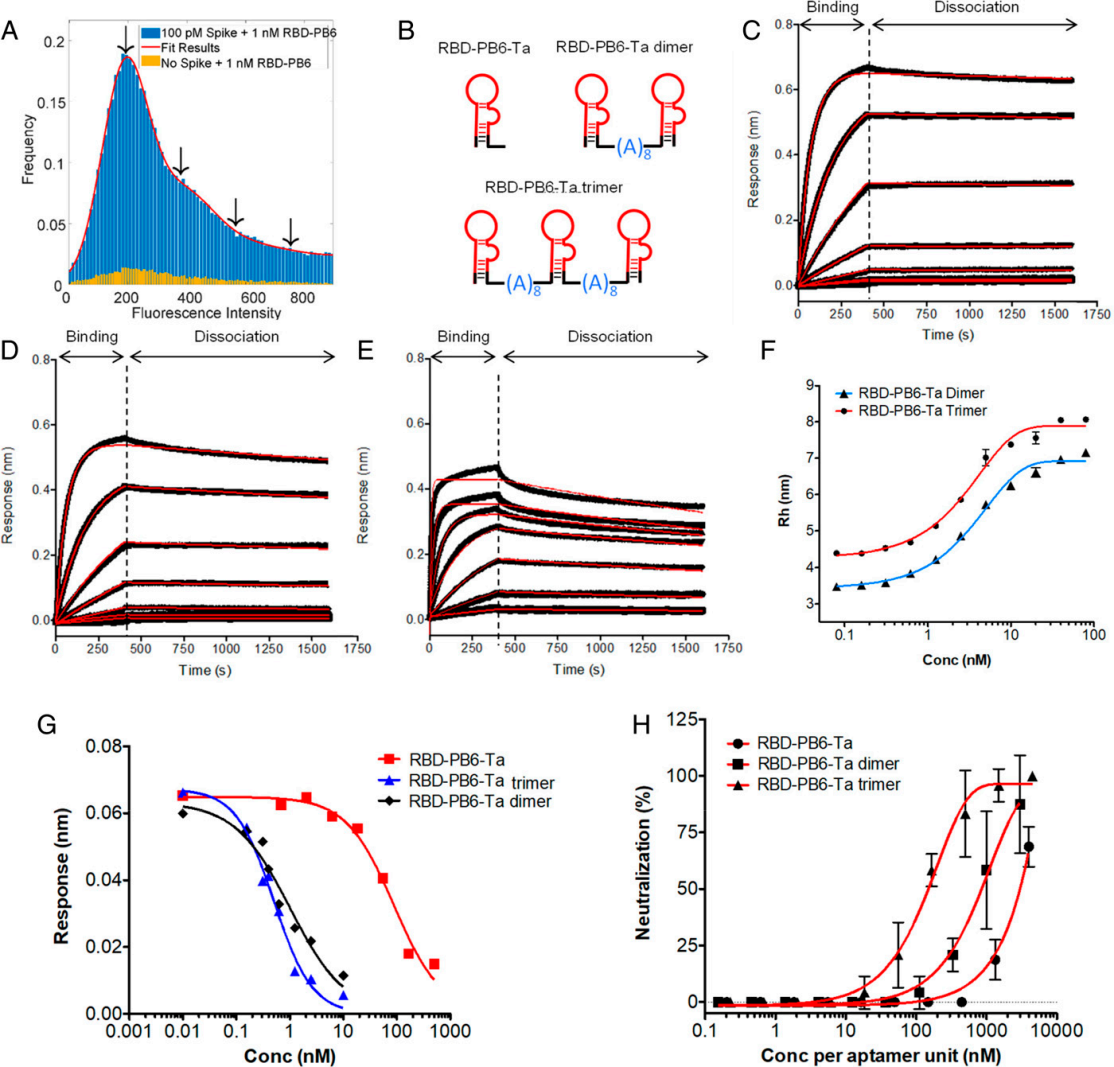
Multivalency enhances aptamer binding and viral neutralization efficiency. (*A*) Histogram shows fluorescence intensity originating from binding events of RBD-PB6 aptamers on trimeric spike. (*B*) Scheme of multimerized versions of RBD-PB6-Ta aptamer joined by poly(A) linkers. Picomolar affinities for the multimerized aptamers were recorded by BLI for (*C*) biotinylated trimer, (*D*) dimer, and (*E*) monomer RBD-PB6-Ta immobilized on the sensor surface at different concentrations of spike trimer ranging from 150 nM in 1/3 serial dilutions for the monomer and 16 nM in 1/3 serial dilutions for trimer and dimer. (*F*) FIDA binding experiments show superior affinity of trimeric and dimeric aptamers for spike protein. (*G*) Competition assays show enhanced binding inhibition of ACE2:spike interaction with the dimer and trimer of RBD-PB6-Ta. (*H*) Neutralization plaque assay with SARS-CoV-2 virus on ACE2- and TMPRSS2-expressing VeroE6 cells.

The SARS-CoV-2 spike protein adopts a trimeric structure in the viral envelope, and we therefore hypothesized that multimerization of RBD-PB6 would further enhance binding to native spike protein (44). We performed single-molecule total internal reflection fluorescence (TIRF) microscopy experiments to determine the binding stoichiometry between monomer RBD-PB6 and trimeric spike. To that end, biotinylated trimeric spike protein was immobilized on a passivated glass surface functionalized with streptavidin and incubated with RBD-PB6 aptamer labeled at the 3′ end with Cy5. The RBD-PB6 aptamer bound to the spike-decorated surface and all binding events, which appeared as bright spots on the detector, were recorded over time (*SI Appendix*, Fig. S6). The fluorescence time traces associated with each spot often showed multiple binding and dissociation events, and several fluorescence intensities appeared in binding events. Analysis of all binding events showed the presence of distinct populations with associated fluorescence intensities matching with the simultaneous presence of one, two, three, or more aptamers interacting with the immobilized spike protein (Fig. 4*A* and *SI Appendix*, Fig. S6). Some of these events may arise from aptamer aggregation in solution. We also saw events with higher fluorescence intensities in control experiments in the absence of the spike protein (yellow bars, Fig. 4*A*). However, these higher intensity events were significantly more frequent in the presence of spike protein, indicating that two and three molecules of RBD-PB6 can simultaneously bind to trimeric spike.

To exploit the increased binding avidity associated with multivalency, we multimerized RBD-PB6-Ta to dimeric and trimeric forms. To determine the optimal linker length, we conducted a trimeric spike–ACE2 competition assay on the BLI platform using DNA scaffolds with linkers ranging from 3 to 27 adenosines (∼1.5 nm to 13.5 nm) and an extended version of the RBD-PB6-Ta aptamer enabling it to hybridize to the different DNA scaffolds. The largest increase in competition efficiency was obtained with linkers between 7 and 11 adenosines. Based on this screening (*SI Appendix*, Fig. S7), we designed dimers and trimers of RBD-PB6-Ta made exclusively of RNA with ∼4-nm A_8_ spacers between each aptamer (Fig. 4*B*). We tested the binding affinity for dimer and trimer versions of RBD-PB6 using BLI by immobilizing the aptamers and adding trimeric spike protein in different concentrations. This showed that the binding of multimerized aptamers was strongly improved, exhibiting affinities in the low picomolar range (K_D_ values of 0.4 nM, 72 pM, and 39 pM for monomer, dimer, and trimer, respectively; Fig. 4 *C–E* and *SI Appendix*, Fig. S8). Analysis of the kinetic rate constants showed fast association rates for all constructs with similar *k_a_* values of 8.6 × 10^5^, 1.0 × 10^6^ and 9.5 × 10^5^ M^−1^s^−1^ for the trimer, dimer and monomer, respectively. Larger differences were observed in the dissociation rate constants, which showed strong avidity effects: The dimer and especially the trimer exhibited very slow dissociation constants with *k_d_* values of 2.17 × 10^−5^ s^−1^ and 7.23 × 10^−5^ s^−1^, respectively, compared to the monomer (Fig. 4*E*; *k_d_* = 1.99 × 10^−4^ s^−1^). When tested for binding to trimeric spike protein using FIDA, we saw a strong enhancement of binding affinity for the multimerized aptamers compared to the monomer, with K_D_ values of ∼0.8 and 1.4 nM for the trimer and dimer (Fig. 4*F*), which enters an affinity range so strong that it cannot be accurately determined by FIDA. The Rh of the dimer and the trimer aptamers (3.4 and 4.3 nm, respectively) increased considerably to 7.3 and 8.2 nm upon binding to the trimeric spike protein, which is in good agreement with the expected values. Our data emphasize how avidity enhances the molecular recognition properties of multivalent aptamers.

The enhanced affinity of multivalent aptamers was next investigated in BLI competition experiments (Fig. 4*G* and *SI Appendix*, Fig. S9). To this end, trimeric spike protein was incubated at a fixed concentration (0.5 nM) with different amounts of aptamers, and the interaction with ACE2 protein immobilized on the sensor surface was measured. The dimeric and trimeric aptamer constructs showed IC_50_ values of 0.67 and 0.35 nM, respectively, which is close to the limit of detection of the assay, whereas the monomeric aptamer showed an IC_50_ value of 47 nM, ∼100-fold higher than the multimerized versions (Fig. 4*G*). Hence the multimerized aptamers show a clearly increased ability to block binding between trimeric spike and human ACE2.

RNA aptamers bearing 2′-fluoro modifications at the pyrimidines are known to be more stable than unmodified RNA and DNA aptamers (36, 37). We determined the serum stability of RBD-PB6, RBD-PB6-Ta, and multimerized versions (dimer and trimer) by incubating the aptamers in either phosphate buffer or in Dulbecco’s Modified Eagle’s medium (DMEM) +10% fetal calf serum (FCS) at 37 °C for different time periods. Visualization of the degradation by polyacrylamide gel electrophoresis revealed that the aptamer constructs remained largely intact for up to 24 h in media supplemented with FCS with a half-life of ∼50 h (*SI Appendix*, Fig. S10), and up to 4 d in phosphate buffer (*SI Appendix*, Fig. S11). This contrasts strongly to nonmodified RNA aptamers that are degraded in serum within minutes. Together with the infectivity studies in cell culture, these results demonstrate that all the RBD-RB6 aptamer variants are stable and functional for extended times in serum.

Finally, to test the ability of RBD-PB6 to neutralize infection by real SARS-CoV-2 virus, we performed plaque reduction neutralization tests using replication competent SARS-CoV-2 infection of ACE2- and TMPRRS2-expressing VeroE6 cells in the presence of monomeric, dimeric, or trimeric aptamer. All three aptamer forms showed a neutralizing effect on SARS-CoV-2 infection, but with a clear enhancement for the dimer and trimer forms (Fig. 4*H*). The trimeric aptamer showed the strongest neutralizing effect, inhibiting SARS-CoV-2 entry with an IC_50_ of 46 nM without apparent cytotoxicity at the highest concentration tested (*SI Appendix*, Fig. S12). The dimeric aptamer showed an about 10 times lower effect, with IC_50_ values of 387 nM, whereas the monomeric aptamer, RBD-PB6 Ta, further declined the neutralizing activity about 10 times to an IC_50_ of ∼1.5 µM to 5 µM. When comparing efficiencies per aptamer unit, dimeric and trimeric aptamers show approximately 5-fold and 25-fold stronger neutralizing effect, respectively, compared to monomeric aptamer (Fig. 4*H*).

### RBD-PB6 Binds with High Affinity to the Alpha and Beta Variants of SARS-CoV-2 Spike Protein.

To evaluate how RBD-PB6 performs against emerging SARS-CoV-2 variants, we performed binding assays using recombinant RBD containing the most prevalent mutations, in particular, those found in the alpha and beta lineages. His-tagged proteins were immobilized onto an NTA-functionalized BLI sensor and subsequently incubated with different concentrations of RBD-PB6.

BLI binding assays showed that RBD-PB6 binds strongly (K_D_ = 38 nM) to RBD carrying the mutations found in variant beta (K417N, E484K, N501Y) (*SI Appendix*, Fig. S13). Moreover, the aptamer binds with strong affinity (K_D_ = 12 nM, *SI Appendix*, Fig. S13*C*) to the alpha variant of the spike S1 subunit. BLI competition experiments further showed that RBD-PB6 and RBD-PB6-Ta also inhibit binding between RBD-beta and ACE2, with IC_50_ values of 577 and 556 nM, respectively (*SI Appendix*, Fig. S13*B*). Therefore, our data confirm that the current and most prevalent mutations on the spike protein do not affect recognition by RBD-PB6.

## Discussion

The coronavirus outbreak has caused a public health emergency with millions of deaths and people infected with severe complications. Moreover, the pandemic has had enormous socioeconomic impact of international concern. Thus, it is of utmost importance to develop new molecules and drugs for fast, reliable, and cheap detection and treatment of the disease.

Herein, we report a chemically stabilized RNA aptamer that binds with high affinity and selectivity to both the RBD alone and the intact spike protein of SARS-CoV-2. Binding affinities were characterized by both surface and solution biophysical techniques. The experimentally determined size of the individual species assessed by FIDA matches the theoretical values. The relatively small increment in size observed upon aptamer binding suggests that RBD-PB6 establishes multiple interactions with spike protein in agreement with a tightly assembled complex.

The presence of RBD-PB6 blocked the interaction between SARS-CoV-2 spike protein and host receptor ACE2 and prevented viral entry in cells, suggesting that the RBD-PB6 and ACE2 binding sites on spike overlap. Multimerization of the aptamer to dimeric and trimeric forms resulted in an ∼10-fold and 100-fold stronger binding affinity, respectively, mirrored by increased inhibitory effect on SARS-CoV-2 infection, with IC_50_ values in the low nanomolar range.

Recently, multiple variants of SARS-CoV-2 virus have emerged and are circulating globally. These variants carry mutations in the spike protein associated with increased transmission, thus leading to an increase in COVID-19 cases (43, 45). In addition, some of these variants contain mutations that could hamper immune detection and weaken Ab-based therapies and vaccine efficiency (46). RBD-PB6 showed high affinity toward spike proteins from the viral lineages alpha and beta, illustrating that the mutations present in spike protein from these variants do not affect RBD-PB6 binding. This contrasts with recent reports showing that Abs raised toward earlier strains of SARS-CoV-2 may exhibit decreased binding to new variants of spike (47). A plausible explanation may be that aptamers tend to recognize larger, often discontinuous, epitopes on the target protein surface and hence may be less sensitive to single amino acid mutations. Eight of the 14 amino acids from SARS-CoV-1 known to interact with ACE2 are conserved in SARS-CoV-2, but there are substantial differences in the binding interface (new salt bridge between Lys417 of SARS-CoV-2 S protein and Asp30 of ACE2, new hydrogen bonding networks, and an increment in hydrophobic contacts). Hence, the binding mode of the aptamer probably involves unique contact/areas or interfaces essential for viral ACE2 recognition that are not shared in SARS-CoV-1.

RBD-PB6 can easily be mass produced in a fast, robust, and reproducible manner using conventional synthetic approaches. This is clearly advantageous when comparing to Ab production that relies on animal immunization and displays low batch-to-batch reproducibility. Moreover, the aptamer bears 2′-fluoro modifications resistant to nuclease degradation, which provides serum stability in connection with therapeutic application, diagnostic testing, and sample distribution. Hence, our aptamer provides a promising lead for COVID-19 treatment and cost-effective detection platforms for the rapid diagnosis of SARS-CoV-2.

## Materials and Methods

### Selection of 2′-Fluoro RNA Aptamers.

All oligonucleotides, including primers and single-stranded DNA library, were synthesized by Integrated DNA Technologies. For the selection of 2′-fluoro RNA aptamers to RBD, the library design previously described (38, 39) was used, with, however, a 36-nt random region (here termed KK-N36 library: 5′-GGATCCATGGGCACTATTTATATCAAC-N36-AATGTCGTTGGTGGCCC). In order to generate a library of double-stranded DNA (dsDNA) transcription templates, we performed a first annealing step of the Forward primer (KKfw36) and the KK-N36 library at 75 °C for 15 min and slowly cooled it to room temperature (RT). Annealed primers were extended by Klenow polymerase (Klenow Fragment, exo-, ThermoScientific). Purification of the dsDNA products was performed with 6% nondenaturing polyacrylamide gel. The initial RNA library was transcribed in a reaction containing dsDNA template (1.7 nmol at 0.7 µM final concentration, *ca*. 10^15^ molecules) and mutant T7 RNA polymerase Y639F in transcription buffer (80 mM Hepes [pH 7.5], 25 mM MgCl_2_, 2 mM spermidine-HCl, 2.5 mM each adenosine 5′-triphosphate, guanosine 5′-triphosphate [Jena Biosciences], and 2′-F-dCTP and 2′-F-dUTP [TriLink Biotechnologies], supplemented with bovine serum albumin [BSA] [heat shock fraction pH 7 ≥ 98%, Sigma-Aldrich], pyrophosphatase inorganic [from yeast] and dithiothreitrol [ThermoScientific]). Reactions were purified on an 8% denaturing polyacrylamide gel and recovered via passive elution and ethanol precipitation.

Selection was performed using His-tagged SARS-CoV-2 RBD spike purified protein (amino acids 319 to 532). Beads were prepared as described by the manufacturer; in the initial selection cycle, 5 µg of WT SARS-CoV-2 RBD spike protein His-tagged and of control His-tagged protein [Ctr-His, Rb17c Nb (48) His-tagged] were incubated with Dynabeads His-Tag Isolation and Pulldown (Invitrogen), using manufactureŕs recommended loading capacity according to the molecular weight of protein, for 30 min at 25 °C with shaking (800 rpm) in 10 mM phosphate buffer pH 7.4, 300 mM NaCl, and 0.01% Tween20. Three washing steps in washing buffer (WB; 10 mM phosphate buffer pH 7.5, 3 mM MgCl_2_, and 150 mM NaCl supplemented with 0.1 mg/mL BSA) were performed. Beads were resuspended in selection buffer (WB supplemented with 0.1 mg/mL salmon sperm DNA [Invitrogen] and 0.1 mg/mL BSA). Library was refolded in WB prior to incubation with the beads as follows: 1) 90 °C for 2 min, 2) 65 °C for 5 min, and 3) 37 °C for 5 min, and left at RT; 1.7 nmol of the starting library was initially incubated at a final volume of 150 µL with the counterselection beads at RT for 45 min and shaking at 700 rpm. Unbound sequences were then transferred to the RBD-modified beads and incubated at the same conditions as before. After three washing steps with WB (0.5 mL, 1 min), beads were subjected to reverse transcription for complementary DNA (cDNA) generation of the bound sequences using the reverse primer (KKrv36) and SuperScript III (Invitrogen). The dsDNA transcription templates for the next selection cycle were generated by PCR using the KKfw36 and KKrv36 primers (see sequences in *SI Appendix*) and Phusion High-fidelity DNA Polymerase (ThermoScientific), followed by BamHi digestion (ThermoScientific) and dsDNA purification with GeneJET PCR purification kit (ThermoScientific). The 2′-fluoro pyrimidine RNA for subsequent cycles was produced by transcription as detailed above and purified on 8% denaturing polyacrylamide gels. In the succeeding cycles, the amount of 2′-fluoro pyrimidine RNA was kept constant at 150 pmol (cycles 2 to 8) while the amount of RBD protein was decreased during selection from 5 µg (cycle 2), 2 µg (cycle 3), 1 µg (cycle 4), and 0.5 µg (cycles 5 to 8). The protein amount used for counterselection was kept constant at 2 µg from selection cycle 2. Incubation time with the counterselection beads was kept at 45 min in all selection cycles, while incubation with target beads was decreased to 15 min in the last selection cycles (from cycle 4). Longer washing steps were performed over the selection in order to increase stringency. From selection cycle 6, an additional counterselection step was introduced after the first counterselection by incubating the nonbound sequences with empty beads for 15 min.

### NGS.

For selection cycles 3 to 8, samples were prepared for NGS analysis on iSeq100 Sequencing System (Illumina). Reverse transcription of the 2′-fluoro pyrimidine RNA libraries of each selection cycle was performed followed by generation of dsDNA template by PCR. After purification of the PCR product as described above, PCR with primers containing the reverse and forward primer sequences combined with a unique index sequence and sequencing adapters was performed, and the different samples containing different indexes were mixed in equimolar amounts (1 µg). To separate the PCR product from the primers, purification of 3.5 µg of the mixed library was performed with the Pippin Prep instrument with a 3% agarose, 100- to 250-bp cassette (Sage Science). Library size and purity was validated on a 2% agarose gel, and the concentration was measured with Qubit fluorimeter (Invitrogen). Final preparation of the library was performed as described by iSEQ100 Sequencing System guide. One hundred and fifty–base pair paired end sequencing was carried out. Raw NGS data were analyzed using an in-house software (49).

### Quantitative PCR Assay.

For qPCR quantification, protein-modified beads were prepared as described above using 0.5 µg of control His-tagged protein (Rb17c Nb) and/or RBD-His or the trimeric stabilized spike protein His-tagged. Incubation with 20 pmol of 2′-fluoro pyrimidine RNA libraries or single clones was performed in a final volume of 100 µL of binding buffer for 30 min at RT and shaking at 700 rpm. Three washing steps in WB were performed (0.3 mL, 1 min), and beads were finally resuspended in 12 µL of deionized water for reverse transcription as described above. One microliter of the generated cDNA was diluted in 74 µL of water for analysis via qPCR. Twenty microliters of diluted sample were added to 20 µL of PCR master mix containing 1× LightCycler 480 SYBR Green I master mix (Roche) and 500 nM KKrv36 and KKfw36 primers. Thermal conditions were optimized to 7 min at 95 °C followed by 40 cycles of 10 s at 95 °C, 20 s at 60 °C, and 30 s at 72 °C. Thermal cycling was performed in a LightCycler 480 (Roche). Each sample was run in technical triplicates. DeltaCt values were defined as the control protein minus target protein Ct values.

### Protein Expression and Purification.

Proteins were expressed as previously described (50) in Expi293 cells (Thermo Fisher Scientific) in different scales using the commercial ExpiFectamine system and subjected to affinity purification.

### Aptamer Production and Purification.

RBD-PB6 aptamer and the truncated and multimerized versions of RBD-PB6 were produced by in vitro transcription as described for the library preparation in the selection of 2′-fluoro aptamers section. RBD-PB6 aptamer template was generated by PCR amplification using the corresponding template with KKfw36 and KKrv36 primers (*SI Appendix*). The double-stranded templates of the truncated and multimerized versions of RBD-PB6 aptamer were produced by annealing of the two cDNA strands (see sequences in *SI Appendix*.

### BLI Binding Assays.

BLI binding experiments were performed in Octet RED96 equipment (ForteBio) and analyzed using either the instrument’s software or Prism (GraphPad Prism 5.0) software. Binding sensorgrams were aligned to dissociation, following subtraction of the reference well/sample and globally fit to a 1:1 binding model. For these binding kinetic experiments, 96-well plates (black, flat bottom, Greiner) were used. In general, orbital shake speeds of 700 rpm or 1,000 rpm were used for BLI experiments.

For aptamer binding assays, different His-tagged spike protein constructs were diluted in binding buffer (10 mM phosphate buffer pH 7.5, 150 mM NaCl, and 3 mM MgCl_2_ with 0.1% BSA and 0.05% Tween-20) at 2.5 µg/mL concentration and immobilized onto Ni-NTA–coated biosensors (OCTET Ni-NTA [NTA] Biosensors, Sartorius). Serial dilutions of the RBD-PB6 aptamer (previously folded in 10 mM phosphate buffer pH 7.5, 150 mM NaCl, and 3 mM MgCl_2_ using a ramp temperature of 1 min at 90 °C, 2 min at 65 °C, 2 min at 37 °C, and, finally, at RT) in binding buffer were prepared for the binding measurements. Baseline was recorded before each binding event (including association, dissociation, and regeneration steps). First, the protein-coated sensor was dipped into the aptamer solution (association step), then into a well containing only buffer (dissociation step). Finally, three cycles of regeneration and cleaning were performed consisting of first dipping the sensor into a glycine solution (10 mM at pH 1.4) and then into buffer for 5 s each. The process was repeated for each aptamer concentration.

Biotinylated aptamers diluted in binding buffer at 40 nM concentration were loaded on streptavidin-coated sensors (OCTET Streptavidin [SA] Biosenors, Sartorius) until 0.15-nm response was reached. Baseline was recorded prior to binding measurements. The binding kinetics were recorded as follows: Eight aptamer-loaded sensors were dipped in 1/3 serial dilutions of spike protein (stabilized trimeric protein with a C-tag provided by ExpreS2ion Biotechnologies) for 400 s during the association step. For the multimerized aptamers (dimer and trimer), the highest concentration started at 16 nM, whereas, for the monomeric RBD-PB6-Ta, the highest concentration was 150 nM. Then, the sensors were moved to only buffer for 1,200 s (dissociation step). Regeneration was achieved by doing three cycles of consecutive steps, first dipping into phosphoric acid (500 mM) and then into binding buffer for 5 s each. Subsequently, the aptamer-loaded sensors were dipped again in freshly prepared serial dilutions of spike protein for another binding measurement (including association and dissociation steps).

For the ACE2 competition assay, Fc-tagged ACE2 protein ([amino acids 19 to 740] expressed in CHO, The Native Antigen Company) at 2.5 µg/mL concentration in binding buffer was captured on a protein G-coated sensor (OCTET ProteinG [ProG] Biosensors, Sartorius) for 300 s or until 0.5-nm response was reached. RBD (homemade), RBD beta variant (AcroBiosystems), S1 alpha variant (SinoBiological), and spike trimer (ExpreS2ion Biotechnologies) proteins were used as ligands at fixed concentrations of 50, 25, and 0.5 nM, respectively. The different aptamer constructs (full-length, truncated, and multimerized versions) were previously folded as described above and incubated at different concentrations with these proteins for 20 min at RT. The octet protocol consisted in dipping the ACE2-loaded sensors first in buffer for 60 s (baseline), then in different solutions of RBD or spike protein plus aptamer for 150 or 600 s, respectively (association), and, finally, in binding buffer again (dissociation) for 150 or 600 s (for RBD or spike, respectively). Regeneration was achieved by doing three cycles of consecutive steps, first dipping into 10 mM Glycine pH 1.4 and then into binding buffer for 5 s each. The process, including the ACE2 loading, was repeated at different aptamer concentrations.

### FIDA.

FIDA experiments were conducted using a FIDA 1 instrument employing light-emitting diode–induced fluorescence detection using an excitation wavelength of 480 nm and emission wavelength of >515 nm (Fida Biosystems ApS). Noncoated capillaries with inner diameter 75 µm, outer diameter 375 µm, total length 100 cm, and length to detection window 84 cm (Fida Biosystems) were applied. Indicator samples were prepared with 25 nM Alexa Fluor 488–labeled RBD-PB6, RBD-PB6-Ta dimer or RBD-PB6 trimer premixed with either dilution series of RBD (0 μM to 2.5 μM) or spike trimer (0 nM to 80 nM) in assay buffer (PBS with 3 mM MgCl_2_ and 0.1% BSA). Analyte samples contained RBD/spike trimer dilution series only. All samples were analyzed using the following procedure. Initially, the capillary was flushed with assay buffer at 3,500 mbar for 120 s and then at 1,500 mbar for 20 s. Indicator samples were subsequently applied at 50 mbar for 10 s followed by analyte samples at 400 mbar for 180 s. The Taylorgrams were interpreted using the FIDA software suite, version 2.04 (Fida Biosystems ApS) with a Taylorgram fraction setting of 75%.

### Biotinylation of Aptamers/Fluorescent Tagging.

Biotinylation and fluorescence labeling of aptamers was performed by 3′-end ribose oxidation using sodium metaperiodate followed by reaction with Alexa Fluor 488–Hydrazide or EZ-link Biotin-LC-Hydrazide using the standard protocol of the provider (Thermo Fisher Scientific). Cy5 hydrazide was obtained from Lumiprobe.

### TIRF Single-Molecule Fluorescence.

Single-molecule TIRF microscopy measurements were recorded using a nanoimager microscope (ONI). Samples were added to a cover slide chamber consisting of a pair of quartz and glass slides assembled together by Parafilm strips. The surface of the coverslip slide was chemically modified with poly-(ethylene glycol) (PEG) and biotinylated PEG (Laysan Bio) at a 160:1 ratio, to reduce nonspecific binding by aptamers and proteins (51).

Solutions containing the spike protein and aptamers were prepared in imaging buffer consisting of 100 mM NaCl, 3 mM MgCl_2_, and 10 mM phosphate buffer (pH 7.4). The PEG passivated sample chamber was first incubated with a solution of 1% wt/vol Tween-20 for 10 min (52), then with a solution of 0.1 mg/mL streptavidin linker protein (Invitrogen) for 7 min to immobilize single biotinylated spike proteins. After each step, the sample chamber was flushed with surface passivating buffer consisting of 0.2% wt/vol Tween-20 and 0.5% wt/vol BSA in imaging buffer. In the next step, the sample chamber was incubated with 100 pM of spike protein for 10 min, and free protein was washed away with surface passivating buffer. Finally, 1 nM Cy5 labeled aptamer was added just before measurements. The solution containing the aptamer also included an oxygen scavenging system (17 U/mL glucose oxidase, 4.5 mg/mL glucose, and 260 U/mL catalase) to prevent photobleaching, as well as 2 mM of the triplet quenching reagent Trolox (Sigma-Aldrich) to minimize photoblinking. Fluorescence movies 10 min long were taken with frames of 100 ms using excitation with a 640-nm laser totally internally reflected at the glass–water interface where the spike proteins were immobilized. The laser power was set to ∼8.5 mW to minimize photobleaching.

Data were analyzed using iSMS software (53), and custom scripts written in MATLAB (Mathworks). Fluorescence spots, where signal in a frame was a minimum of 150 counts higher than the background, were identified as possible binding events. Background corrected fluorescence time traces were determined for each spot (54); these showed both on and off events, which were identified using hidden Markov modeling analysis (*SI Appendix*, Fig. S6). Above-background fluorescence intensity for each frame (on events) were plotted in histograms. The data were normalized by the total number of events (both on and off events) per video to represent the average number of on events per field of view and enable comparing different datasets together.

### Neutralization Assay Using Pseudotyped VLPs.

VLPs pseudotyped with spike protein of MERS-CoV, SARS-CoV-1, and SARS-CoV-2 were used to assess aptamer specificity. For production of VLPs, HEK293T cells were seeded at density of 50.000 cells/cm^2^. The next day, media was exchanged prior to transfection of cells with three plasmids for lentiviral vector packaging gag-pol, REV, and EGFP reporter (kindly provided by Jacob Giehm Mikkelsen, Aarhus University, Aarhus, Denmark) together with a plasmid coding for either MERS (VG40069‐G‐N, Sino Biological), SARS-CoV-1 (kindly provided by Ralf Wagner, University of Regensburg, Regensberg, Germany), SARS-CoV-2 WT (a gift from Zhaohui Qian, NHC Key Laboratory of Systems Biology of Pathogens, Institute of Pathogen Biology, Chinese Academy of Medical Sciences and Peking Union Medical College, Beijing, China [Addgene plasmid #145780]) (55) or SARS-CoV-2 D614G mutant (a gift from Hyeryun Choe, Perlmutter Laboratory, Children's Hospital, Boston, MA and Michael Farzan, Partners AIDS Research Center, Brigham and Women's Hospital, Cambridge, MA [Addgene plasmid #156421]) (56) spike protein using calcium phosphate precipitation. Forty-eight hours posttransfection, supernatant was harvested and filtered through 0.45-μm-pore-sized filter and spiked with 6 mM MgCl_2_. Harvested VLPs were incubated with an aptamer of interest for 90 min at RT prior to transduction. For transduction, HEK293T cells transfected with ACE2 (a gift from Hyeryun Choe [Addgene plasmid #1786]) (4) and TMPRSS2 (a gift from Roger Reeves, Johns Hopkins University School of Medicine, Baltimore, MD [Addgene plasmid #53887]) (57) spiked with Polybrene (6 μg/mL) seeded 10,000 cells per well on a 96-well plate were added VLPs. The next day, media was exchanged; 4 d posttransduction, cells were analyzed for EGFP fluorescence by fluorescence microscopy (IX73 inverted microscope, Olympus equipped with DP73 camera, Olympus) and flow cytometry (Cytoflex, Beckman Coulter).

### Plaque Reduction Neutralization Test with SARS-CoV-2 Virus.

SARS-CoV-2, Freiburg isolate, FR-4286 (kindly provided by Georg Kochs, University of Freiburg, Freiburg im Breisgau, Germany) was propagated in VeroE6 expressing cells expressing human TMPRSS2 (VeroE6-hTMPRSS2) (kindly provided by Stefan Pöhlmann, University of Göttingen, Göttingen, Germany) with a multiplicity of infection of 0.05. Supernatant containing new virus progeny was harvested 72 h postinfection, and concentrated on 100-kDa Amicon ultrafiltration columns (Merck) by centrifugation for 30 min at 4,000 × *g*. Virus titer was determined by median tissue culture infectious dose (TCID50) assay and calculated by the Reed–Muench method. Aptamers were prepared in serial dilutions in DMEM (Gibco) + 2% FCS (Sigma-Aldrich) + 1% Pen/Strep (Gibco) + L-Glutamine (Sigma-Aldrich) + 3 mM MgCl_2_ mixed with SARS-CoV-2 at a final titer of 100 TCID50 per well, and incubated at RT for 1.5 h. “No aptamer” and a “no virus” (uninfected) control samples were included. The virus:aptamer mixtures were added to 2 × 10^4^ Vero E6 TMPRSS2 cells seeded in flat-bottom 96-well plates, and incubated for 12 h in a humidified CO_2_ incubator at 37 °C, 5% CO_2_, before washing off the cells and reincubating for 60 h. The cells were fixed with 5% formalin (Sigma-Aldrich) and stained with crystal violet solution (Sigma-Aldrich). The plates were read using a light microscope (Leica DMi1) with camera (Leica MC170 HD) at 4× magnification, and the cytopathic effect was scored.

### Cytotoxicity MTT Assay.

MTT (3-[4,5-Dimethylthiazol-2-yl]-2,5-diphenyltetrazolium bromide) assay was performed in order to determine the cytotoxicity of the aptamer variants tested in the plaque reduction neutralization test with SARS-CoV-2 virus. Briefly, Vero E6 TMPRSS2 cells (2 × 10^4^ cells per well) were seeded in flat-bottom 96-well plates. Cells were treated with different concentrations of RBD-PB6-Ta trimer aptamer and incubated for 12 h in a humidified CO_2_ incubator at 37 °C, 5% CO_2_, before washing off the cells and reincubating for 60 h. Then, 10 µL of MTT reagent (5 mg/mL) was added in each well and incubated for 30 min in a 5% CO_2_ incubator for cytotoxicity. After incubation, 100 µL of dimethyl sulfoxide was added and incubated on a rocking shaker for 30 min at RT. Finally, absorbance was read at 590 nm.

### Stability Studies.

Two micromoles RBD-PB6-Ta in its monomeric, dimeric, or trimeric form were incubated in DMEM (Gibco) + 10% FCS (Sigma-Aldrich) or PBS at 37 °C. Samples were collected at different time points (0, 1, 3, 6, 8, 24, 32, 48, 96, and 144 h for serum stability samples and 0, 24, 48, 96, and 144 h for the samples incubated in WB). All aliquots were diluted 1:5 in milliQ water and 2:5 in loading dye and run on an 8% denaturing polyacrylamide gel. Gels were quantified using the ImageJ software (https://imagej.nih.gov/ij/), and aptamer half-life was determined using GraphPad Prism software.

## Supplementary Material

Supplementary File

## Data Availability

All study data are included in the article and/or *SI Appendix*.
