# Supplementary material for: A serum-stable RNA aptamer specific for SARS-CoV-2 neutralizes viral entry
Source: Proc Natl Acad Sci U S A. 2021 Dec 7;118(50):e2112942118. doi: 10.1073/pnas.2112942118 (PMC8685691; doi:10.1073/pnas.2112942118)
Supplement: Supplementary File [file pnas.2112942118.sapp.pdf]

PNAS
www.pnas.org

Supplementary Information for
A serum-stable RNA aptamer specific for SARS-CoV-2 neutralizes viral entry

Julián Valero* †, [a], Laia Civit†, [a], Daniel M. Dupont[a], Denis Selnihhin[b], Line Reinert[c], Manja Idorn[c], Brett A. Israels[a], Aleksandra M. Bednarz[a], Claus Bus[a], Benedikt Asbach[d], David Peterhoff[d], Finn S. Pedersen[b], Victoria Birkedal[a], Ralf Wagner[d], Søren R. Paludan[c] and Jørgen Kjems*,[a,b]

Julián Valero and Jørgen Kjems
Email: jvalero@inano.au.dk, jk@mbg.au.dk

This PDF file includes:

Figures S1 to S13
List of templates

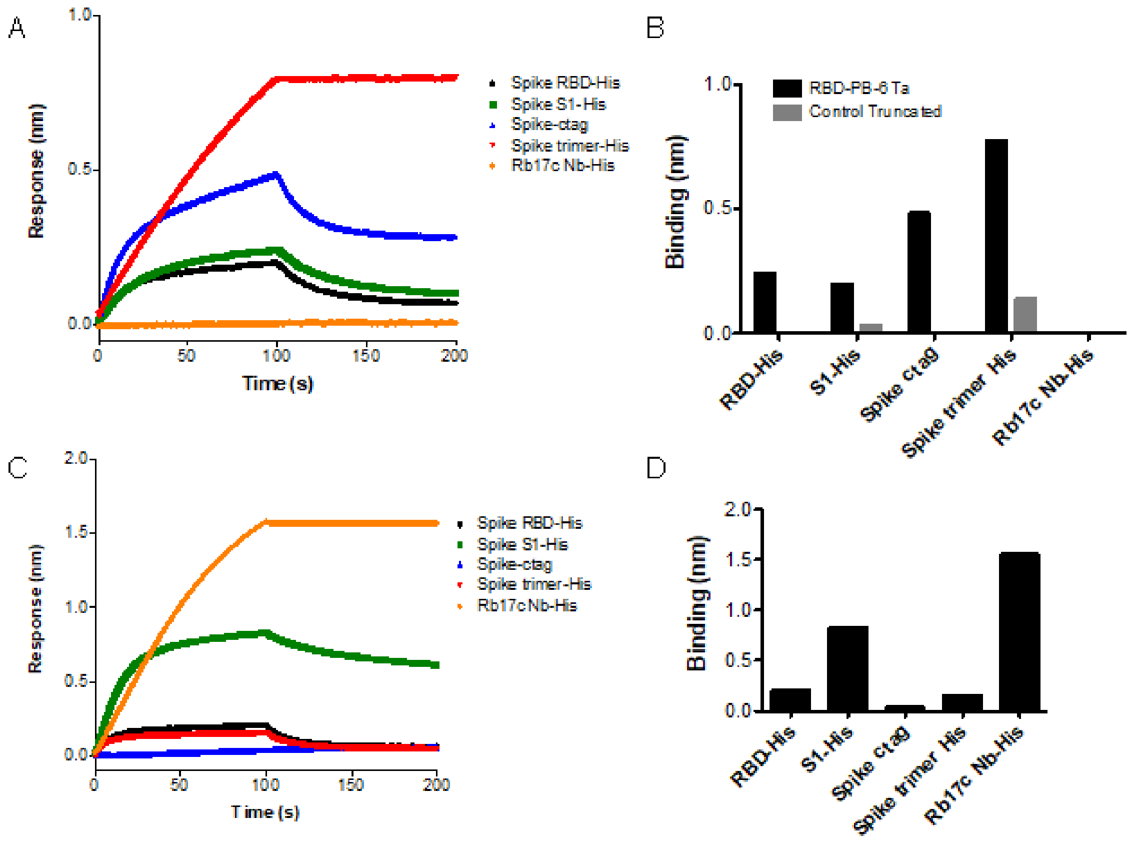

Fig. S1. RBD-PB6 recognizes spike protein in a tag-independent manner. A and B) BLI response of RBD-PB6 and RBD-PB6 Ta immobilized on the surface and incubated with different spike protein constructs: RBD-His (90 nM, black), S1-His (75 nM), spike ctag (150 nM), spike trimer-His (50 nM), nanobody (negative control, orange, 250 nM). C and D) BLI response of a previously reported DNA aptamer (CoV2-RBD-1C) immobilized on the surface and incubated with different spike protein constructs: RBD-His (125 nM, black), S1-His (80 nM), spike ctag (150 nM), spike trimer-His (50 nM), nanobody (negative control, orange, 200 nM).

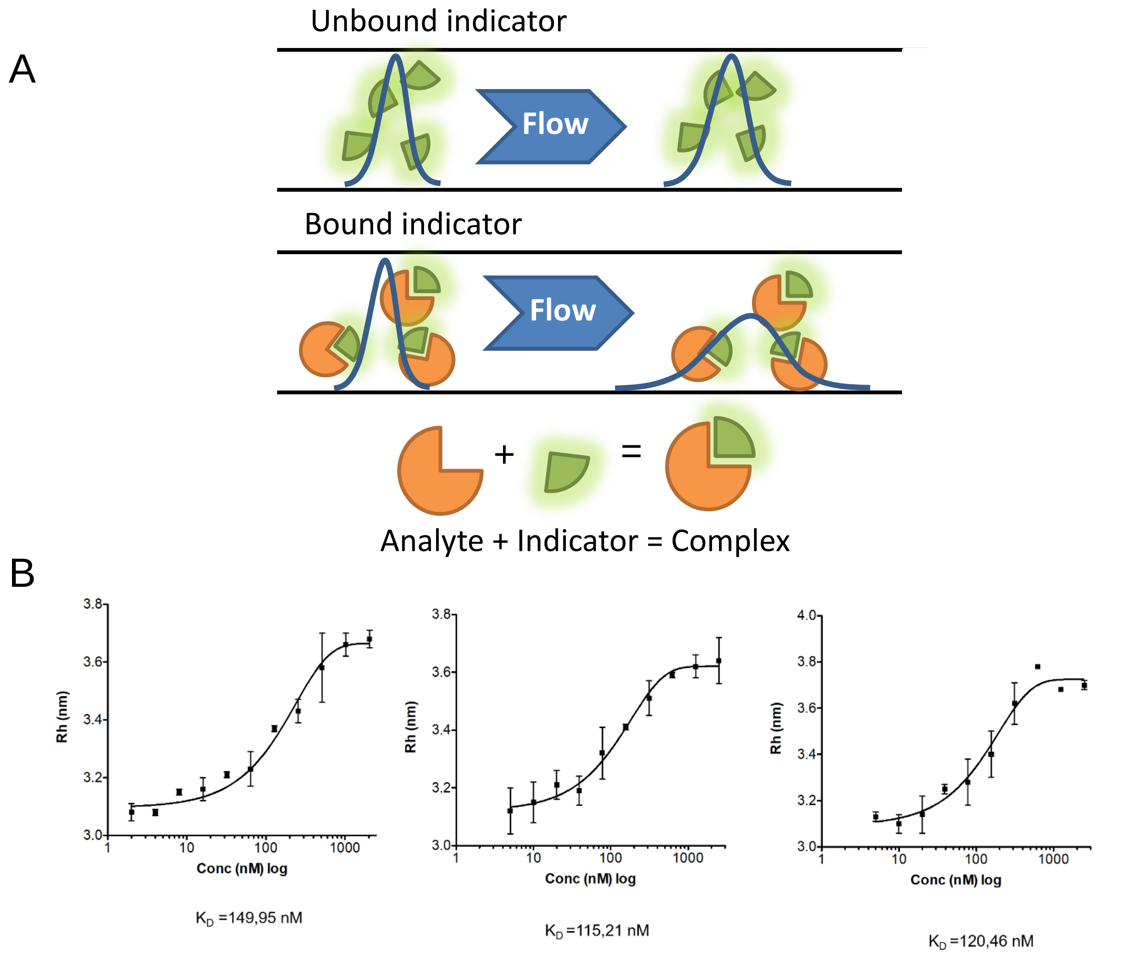

Fig. S2. Flow-Induced Dispersion Analysis determines complex formation and size. A) The principle behind FIDA. Samples are measured in a capillary under hydrodynamic flow. Complex formation changes the diffusivity (apparent size) of the labelled indicator in the dispersion analysis. B) FIDA experiments showing the increase in hydrodynamic radius in solution upon binding of the RBD to the fluorescently labelled RBD-PB6 (black line indicates fitting 1:1 model used).

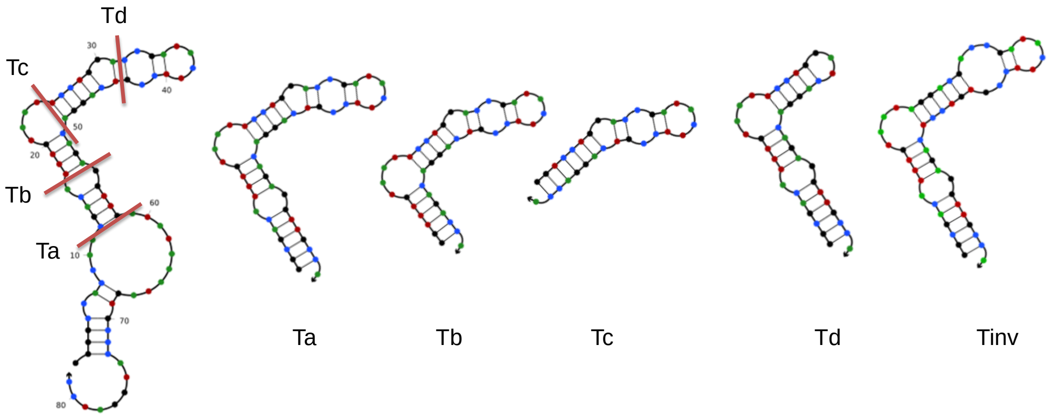

	truncation sequences
**RBD-PB6**	GGGGCCACCAACGACAUUUGUAAUUCCUGGACCGAUACUUCCGUCAGGACAGAGGUUGAUAUAAAUAGUGCCCAUGGAUCC
**RBD-PB6a**	GGCGACAUUUGUAAUUCCUGGACCGAUACUUCCGUCAGGACAGAGGUUGCCA
**RBD-PB6b**	GGUUUGUAAUUCCUGGACCGAUACUUCCGUCAGGACAGACCA
**RBD-PB6c**	GGUCCUGGACCGAUACUUCCGUCAGGACCA
**RBD-PB6d**	GGCGACAUUUGUAAUUCCUGGA------------UCAGGACAGAGGUUGCCA
**RBD-PB6inv**	GGCGACAUUUGUAAU**AGGAC**GACCGAUACUUCCGU**GUCCU**CAGAGGUUGCCA

Fig. S3. RBD-PB6 truncated constructs. NUPACK analysis of secondary structures and sequences and table containing the sequences of each truncated aptamer version. Sequences highlighted in red are the conserved aptamer regions from the full lenght RBD-PB6 aptamer. Mutated nucleobases for the base-pair covariation construct are highlighted in blue.

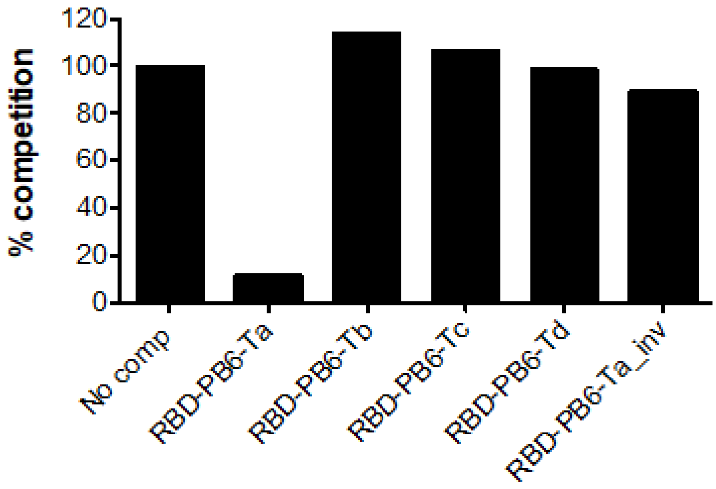

Fig. S4. BLI competition screening experiment of the different truncated versions of RBD-PB6. Competition experiments between RBD and ACE2 was performed using 50 nM RBD-ctag and 1000 nM of each truncated aptamer.

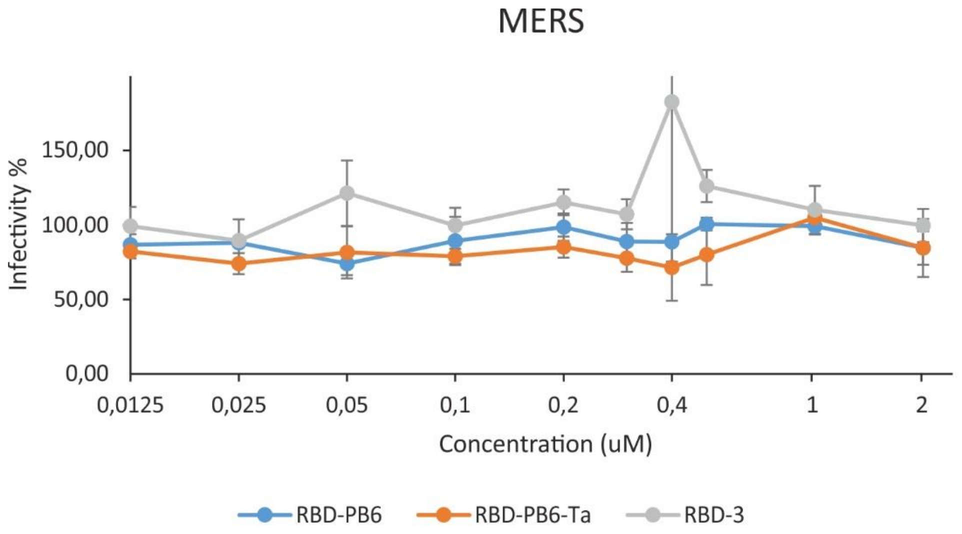

Fig. S5. RBD-PB6 does not block infection by MERS-pseudotyped VLPs in cell culture. VLP neutralization control experiment with MERS pseudotyped virus and RBD-PB6 (blue), RBD-PB6-Ta (orange) and RBD-3 (negative control, grey).

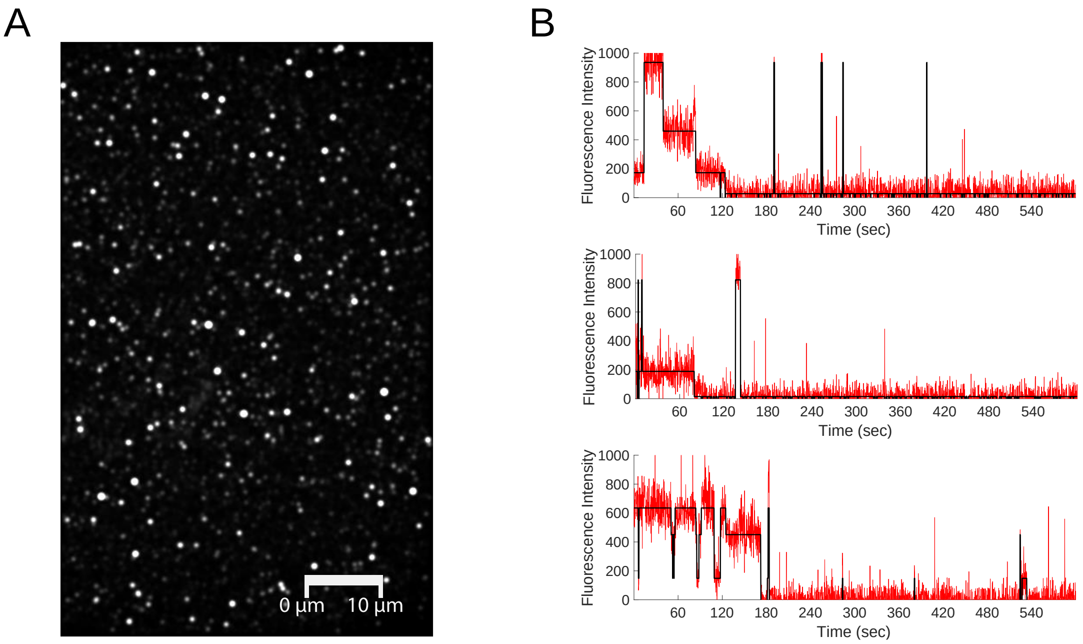

Fig. S6. A) A tiff-stack from the Oxford Nanoimager (ONI) displaying all single-molecule fluorescent events over ten minutes measurements as bright spots where a labelled RBD-PB6 aptamer is bound or close to the imaging surface with low concentration of trimer Spike protein on the surface. The area of the image is 50 µm × 80 µm. B) Examples of single-molecule trajectories showing binding of labelled RBD-PB6 in the presence of trimeric Spike protein as a function of time. The raw data measurements (red lines) are idealized by hidden Markov modeling (black lines) and display multiple stepwise binding/unbinding events in addition to longer, stable binding events.

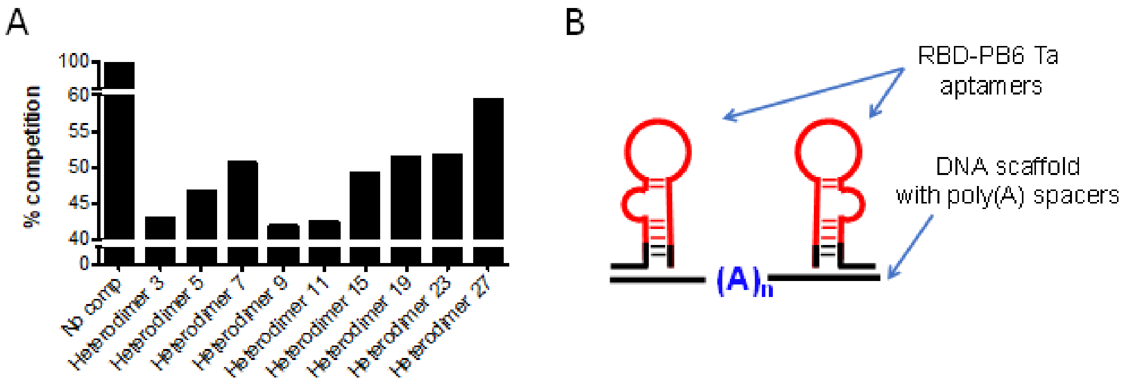

Fig. S7. Screening for the optimal spacer length for RBD-PB6 multimerization. To that end, a DNA scaffold with two docking sites and a variable number of adenosine spacers (number indicated in the bar graph) was used in combination with two equivalents of a truncated RBD-PB6 with an extension to allow hybridization to the scaffold. A) BLI competition experiments showing the percentage of binding inhibition between ACE2 and spike protein (trimer at 7.5 nM) with the different dimeric constructs at 100 nM. The numbering corresponds to the number of adenosines spacing each of the aptamer domains. B) Schematic representation of the dimeric scaffolding used for these screening experiments.

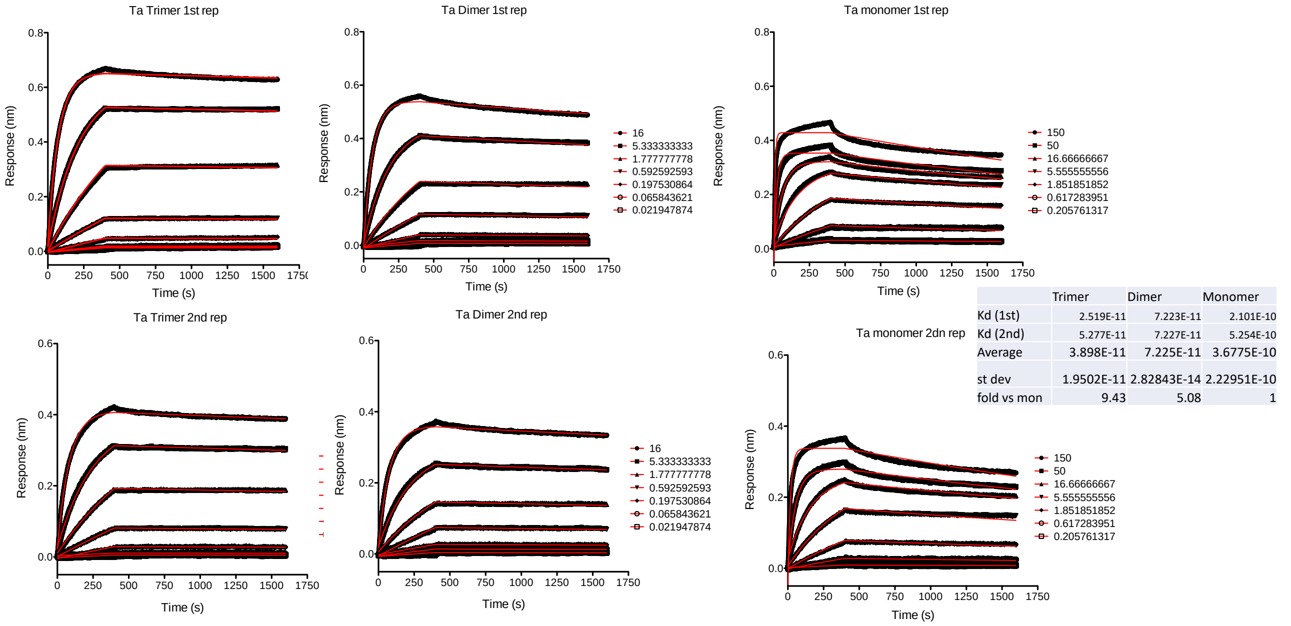

	Trimer	Dimer	Monomer
Kd (1st)	2.519E-11	7.223E-11	2.101E-10
Kd (2nd)	5.277E-11	7.227E-11	5.254E-10
Average	3.898E-11	7.225E-11	3.6775E-10
st dev	1.9502E-11	2.82843E-14	2.22951E-10
fold vs mon	9.43	5.08	1

Fig. S8. BLI experiments shows an increased binding affinity for multimerised RBD-PB6. Biotinylated trimer, dimer and monomer RBD-PB6-Ta were immobilized on a streptavidin-coated sensor at different concentrations of spike trimer ranging from 150 nM in 1/3 serial dilutions for the monomer and 16 nM in 1/3 serial dilutions for trimer and dimer.

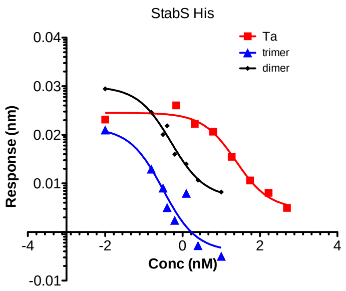

	Ta	trimer	dimer
IC50	23.86	0.3130	0.5044

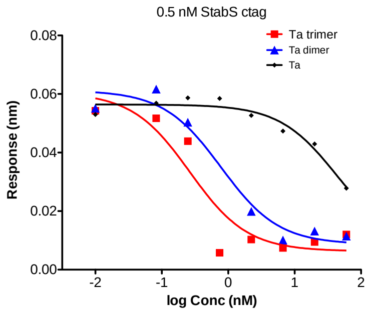

	Ta trimer	Ta dimer	Ta
IC50	0.2636	0.7804	43.33

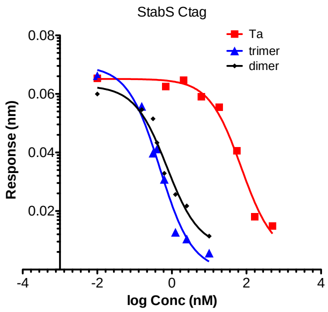

	Ta	trimer	dimer
IC50	72.53	0.4780	0.7329

	trimer	dimer	monomer
IC50 (Ctag1st)	0.2636	0.7804	43.33
IC50 (Ctag2nd)	0.478	0.7329	72.53
IC50 (Stab-His)	0.313	0.5044	23.86
Average	0.35	0.67	46.57
st dev	0.11	0.15	24.50
fold vs mon	132.486250	69.2471626	1

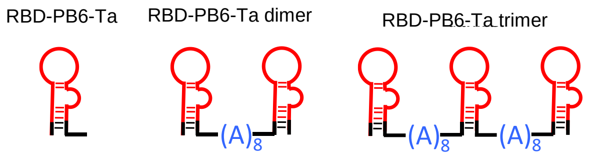

Fig. S9. Multimerised RBD-PB6 effectively blocks the interaction between ACE2 and full-length, trimeric spike. BLI competition experiments with ACE2-Fc immobilized on a G-protein coated sensor and subsequenly dipped in different solutions of trimeric spike protein (0.5 nM) preincubated with increasing concentrations of RBD-PB6-Ta monomer, dimer and trimer.

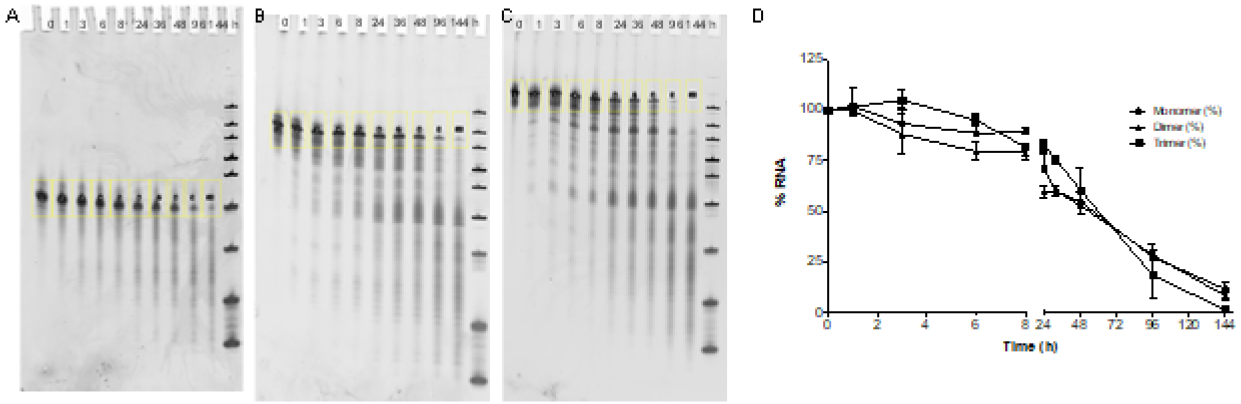

Fig. S10. Time course experiments of aptamer degradation in serum-containing medium. PAGE gel showing the monomer (A), dimer (B) and trimer (C) aptamer incubated in DMEM+10% FCS at 37C at different time points. D) Monomer, dimer and trimer showed half-life values of 51 h, 48 h and 132 h, respectively.

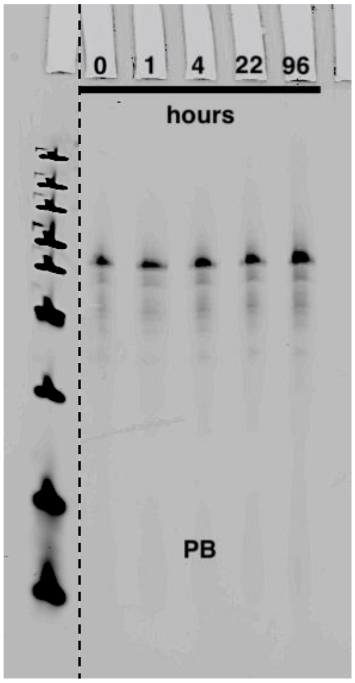

Fig. S11. Time course experiments of aptamer stability in PBS. PAGE gel showing the monomeric aptamer (RBD-PB6) incubated in PBS at 37C at different time points.

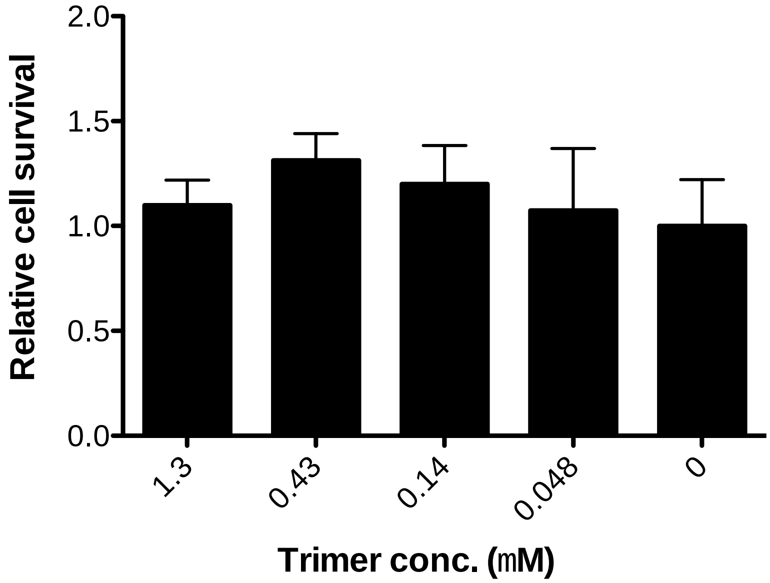

Fig. S12. MTT cytotoxicity studies of trimeric RBD-PB6 ta aptamer. Vero E6 cells were incubated at increasing concentrations of RBD-PB6 ta trimer in the absence of SARS-CoV-2 virus under the same conditions used for the plaque reduction assay. No apparent decrease in cell viability was observed even at the highest aptamer concentrations.

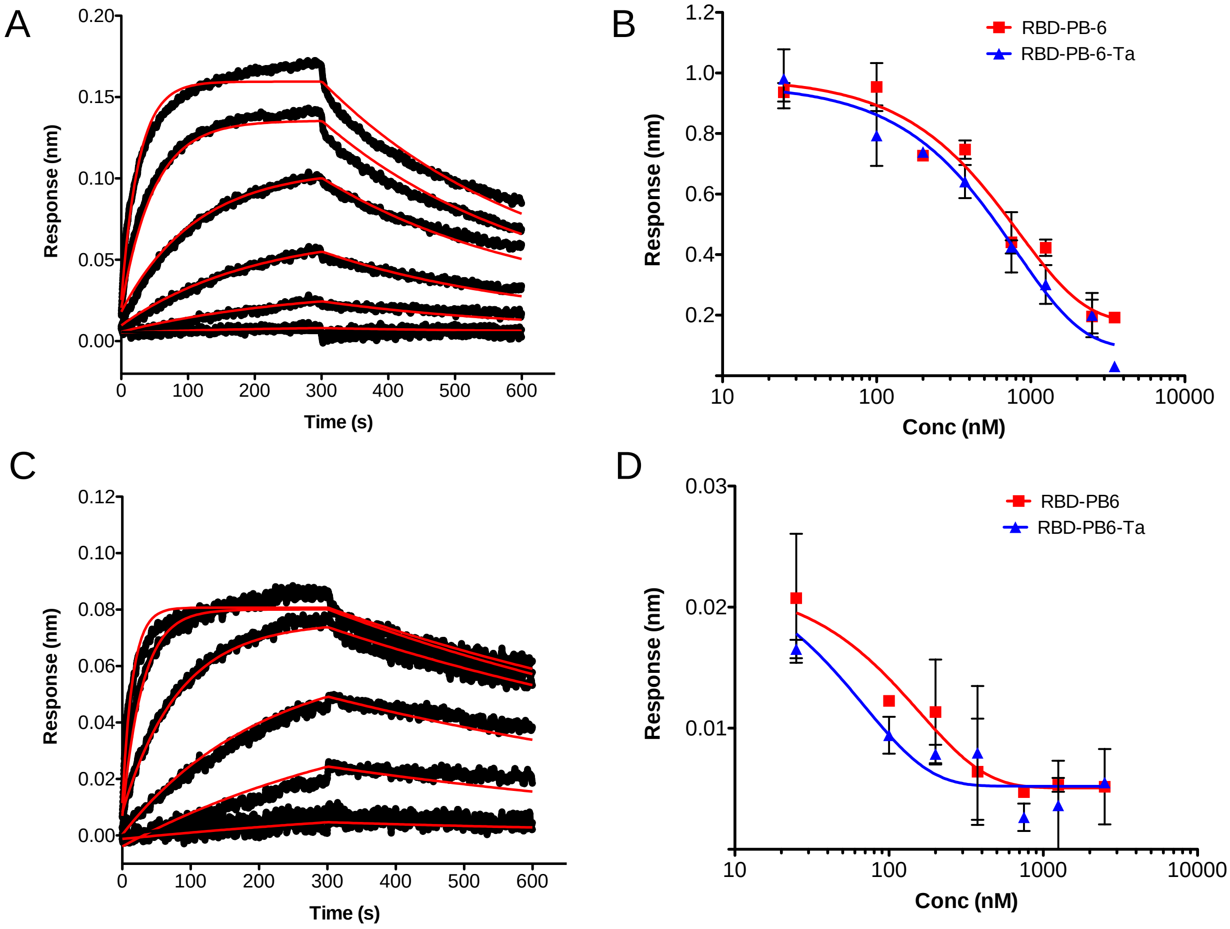

Fig. S13. RBD-PB6 binds to the new variants of SARS-CoV2 spike protein. A) BLI data for RBD-PB6 binding to RBD carrying K417N, E484K and N501Y mutations (present in beta lineage). B) BLI competition experiment between ACE2 and the B.1.351 RBD variant with increasing concentrations of RBD-PB6 and RBD-PB6-Ta. C) BLI data for RBD-PB6 binding to S1 subunit of spike carrying mutations present in alpha lineage. D) BLI competition experiment between ACE2 and the alpha S1 spike subunit variant with increasing concentrations of RBD-PB6 and RBD-PB6-Ta.

List of templates for RBD-PB6, RBD-PB6 ta and multimerized versions:

RBD-PB6a:
TCCTAATACGACTCACTATAGGCGACATTTGTAATTCCTGGACCGATACTTCCGTCAGGACA
GAGGTTG-CCA

RBD-PB6a_C:
TGGCAACCTCTGTCCTGACGGAAGTATCGGTCCAGGAATTACAAATGTCGCCTATAGTGAGT
CGTATTAGGA

RBD-PB6a dimer:
TCCTAATACGACTCACTATAGGGCGACATTTGTAATTCCTGGACCGATACTTCCGTCAGGAC
AGAGGTTGCCAAAAAAAAGGCGACATTTGTAATTCCTGGACCGATACTTCCGTCAGGACAGA
GGTTGCCA

RBD-PB6a dimer_C:
TGGCAACCTCTGTCCTGACGGAAGTATCGGTCCAGGAATTACAAATGTCGCCTTTTTTTTGG
CAACCTCTGTCCTGACGGAAGTATCGGTCCAGGAATTACAAATGTCGCCCTATAGTGAGTCG
TATTAGGA

RBD-PB6a trimer:
TCCTAATACGACTCACTATAGGGCGACATTTGTAATTCCTGGACCGATACTTCCGTCAGGAC
AGAGGTTGCCAAAAAAAAGGCGACATTTGTAATTCCTGGACCGATACTTCCGTCAGGACAGA
GGTTGCCAAAAAAAAGGCGACATTTGTAATTCCTGGACCGATACTTCCGTCAGGACAGAGGT
TGCCA

RBD-PB6a trimer_C:
TGGCAACCTCTGTCCTGACGGAAGTATCGGTCCAGGAATTACAAATGTCGCCTTTTTTTTGG
CAACCTCTGTCCTGACGGAAGTATCGGTCCAGGAATTACAAATGTCGCCTTTTTTTTGGCAA
CCTCTGTCCTGACGGAAGTATCGGTCCAGGAATTACAAATGTCGCCCTATAGTGAGTCGTAT
TAGGA

RBD-PB6:
GAT CCA TGG GCA CTA TTT ATA TCA ACC TCT GTC CTG ACG GAA GTA TCG GTC CAG
GAA TTA CAA ATG TCG TTG GTG GCC C

KKfor (Forward primer): 5'-
CGCGGATCCTAATACGACTCACTATAGGGGCCACCAACGACATT-3'

KKrev (Reverse primer): 5'-CCCGACACCCGCGGATCCATGGGCACTATTTATATCAA-3'
